# Acute Anterior ST-Elevation Myocardial Infarction With Right Bundle Branch Block Complicated by Complete Atrioventricular Block and Cardiogenic Shock in a 21-Year-Old Man: A Case Report

**DOI:** 10.7759/cureus.117374

**Published:** 2026-10-02

**Authors:** Nicholas Wijaya, Hanif A Sulistya, Yusuf Arifin, Arief B Santosa, Fazel N Lucius

**Affiliations:** 1 Emergency Department, Dr. Soepraoen Army Hospital, Malang, IDN; 2 Cardiology and Vascular Medicine Department, Dr. Soepraoen Army Hospital, Malang, IDN; 3 Medical School, Universitas Sumatera Utara, Medan, IDN

**Keywords:** acute myocardial infarction, cardiogenic shock, complete atrioventricular block, percutaneous coronary intervention, right bundle branch block

## Abstract

Acute ST-elevation myocardial infarction (STEMI) is uncommon in very young adults and may initially present with atypical symptoms, resulting in delayed recognition. Severe complications, such as cardiogenic shock and advanced conduction disturbances, can still occur despite limited conventional cardiovascular risk factors. A 21-year-old man initially presented with epigastric pain and was treated for a presumed gastrointestinal disorder. Further evaluation with electrocardiography later revealed acute anterior STEMI. On admission, he was hypotensive and bradycardic, with cardiogenic shock, complete atrioventricular block (CAVB), and right bundle branch block (RBBB). Coronary angiography demonstrated total occlusion of the left anterior descending artery. A PACE 101 H temporary transvenous pacemaker (Osypka Medical, Berlin, Germany) was inserted before primary percutaneous coronary intervention. Revascularisation using a GuReater CoCr Sirolimus-eluting Coronary Stent System (Lepu Medical Technology (Beijing) Co., Ltd., Beijing, China) successfully restored TIMI grade 3 flow. Following reperfusion, hemodynamic status and atrioventricular conduction improved rapidly, allowing pacemaker removal without permanent pacing. This case shows that extensive anterior STEMI can still occur in very young adults despite limited conventional cardiovascular risk factors. Delayed diagnosis may lead to severe complications, including cardiogenic shock and CAVB. However, timely reperfusion may allow recovery of ischemia-related conduction disturbances without permanent pacemaker implantation.

## Introduction

Acute ST-elevation myocardial infarction (STEMI) in young adults (aged ≤35 years) is a rare clinical entity, accounting for only a small fraction of all acute myocardial infarction cases. Unlike older populations, very young patients often lack traditional cardiovascular risk factors, and their initial clinical presentation can be atypical, which frequently leads to delayed recognition [1]. In anterior STEMI, proximal occlusion of the left anterior descending (LAD) artery may also affect the interventricular septum, potentially leading to profound conduction abnormalities.

Right bundle branch block (RBBB) and complete atrioventricular block (CAVB) are uncommon complications in anterior STEMI. While CAVB is more commonly associated with inferior myocardial infarction due to reversible ischemia, its occurrence in anterior STEMI is typically caused by irreversible, extensive necrosis of the infranodal conduction system [2]. Consequently, these conduction blocks in anterior STEMI are generally associated with larger infarct size, pump failure, and much poorer clinical outcomes [3,4].

The combination of anterior STEMI, cardiogenic shock, and advanced conduction disturbance in a very young patient is particularly rare. Previous literature has documented isolated cases of premature STEMI, as well as anterior myocardial infarction complicated by CAVB [1,5]. However, the simultaneous occurrence of extensive anterior STEMI, Killip Class IV cardiogenic shock, CAVB, and RBBB in a 21-year-old patient is exceptionally uncommon and represents the key novelty of this report.

We present this case, reported in accordance with the SCARE 2023 guidelines, to highlight the critical need for early electrocardiographic (ECG) screening in young adults with unexplained atypical symptoms. Furthermore, this report aims to emphasize the potential for rapid conduction recovery following prompt revascularization and to discuss the diagnostic challenges in identifying the precise underlying etiology of premature coronary occlusion when exhaustive evaluations are limited by the hyperacute presentation.

## Case presentation

A 21-year-old man with no known medical history initially presented to a tertiary hospital on 13 January 2026 with epigastric pain accompanied by nausea, dry cough, and generalized weakness. He received symptomatic treatment with intravenous proton pump inhibitor and antiemetic therapy because the symptoms were initially thought to be gastrointestinal in origin. Given his young age and the absence of typical chest pain, acute coronary syndrome was not suspected at presentation. However, as the symptoms worsened, electrocardiography (ECG) was performed and showed ST-elevation myocardial infarction (STEMI). The patient was then referred to our hospital for further management.

Upon arrival, he was fully conscious (GCS E4M6V5) but haemodynamically unstable, with blood pressure 80/50 mmHg, heart rate 47 beats/minute, respiratory rate 28 breaths/minute, oxygen saturation 92% on room air, and temperature 36°C. His body mass index was 29.3 kg/m². Vasopressor therapy was started because of cardiogenic shock (Killip class IV). Physical examination demonstrated cold extremities with delayed capillary refill, while cardiac and pulmonary examinations were otherwise unremarkable. The patient denied any previous episode of severe or typical chest pain. He had smoked approximately one pack of cigarettes daily since the age of 16 years and reported a family history of coronary artery disease in a second-degree relative. Alcohol consumption and illicit drug use were denied. Due to resource limitations and the hyperacute nature of the presentation, comprehensive etiological screenings for premature coronary thrombosis, such as inherited thrombophilia, lipoprotein(a), or autoimmune panels, were not performed.

Because of profound hemodynamic instability and Killip Class IV cardiogenic shock, the patient was immediately resuscitated. Vasopressor and inotropic support were initiated using continuous intravenous infusions of dopamine (5-20 mcg/kg/min) and norepinephrine (0.05 mcg/kg/min). The initial laboratory results were notable for a markedly elevated lactate level of 600. The patient received a loading dose of aspirin (320 mg) and clopidogrel (300 mg), followed by maintenance doses of 80 mg and 75 mg, respectively. High-dose atorvastatin (80 mg) was also administered, later reduced to a 40 mg maintenance dose. Additional adjunctive medical therapy included subcutaneous enoxaparin (60 mg twice daily), intravenous omeprazole (40 mg twice daily), and intravenous ceftriaxone (1000 mg three times daily).

ECG demonstrated ST-segment elevation in leads V1-V4 with reciprocal inferior ST-segment depression. Atrioventricular dissociation with a slow ventricular escape rhythm was consistent with CAVB, accompanied by RBBB morphology (Figure 1). Troponin I level was markedly elevated at 50,000 ng/L, while other laboratory findings were largely unremarkable (Table 1). The patient was diagnosed with acute anterior STEMI complicated by cardiogenic shock, CAVB, and RBBB.

**Figure 1:**
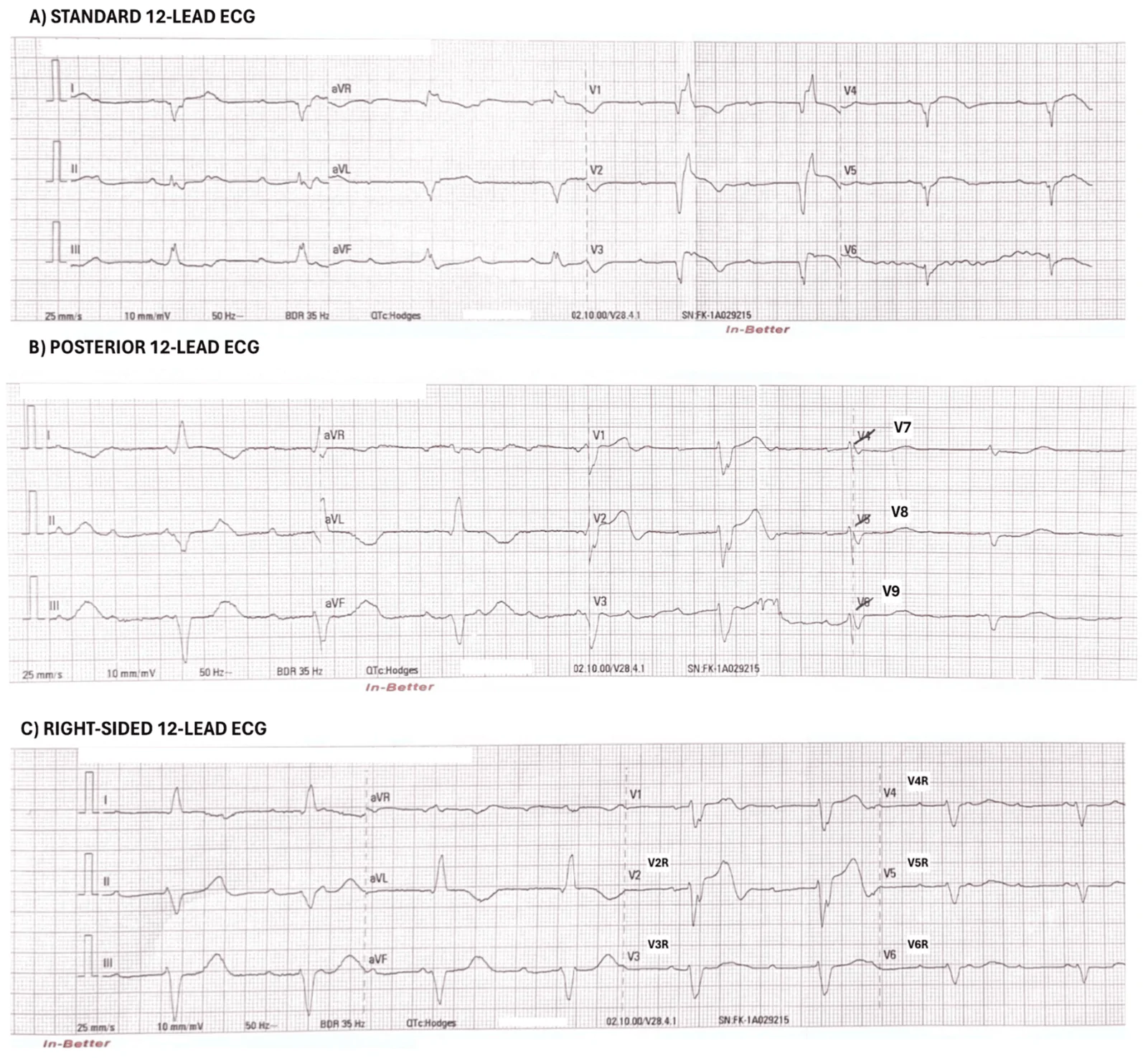
Electrocardiogram at initial presentation showing anterior ST-segment elevation with right bundle branch block and complete atrioventricular block.

**Table 1:** Serial laboratory findings at initial presentation and on admission to our hospital

Parameter (unit)	Normal range	At referring hospital	On admission
Hb (g/dL)	11.5-16.0	11.9	12.6
WBC (cell/μL)	4000-11000	10,520	12,650
Diff count (%):			
Eosinophil	2-4	4	-
Basophil	0-1	0	-
Neutrophil	50-70	75	76
Lymphocyte	25-40	16	17
Monocyte	2-8	5	7
Platelet count (*10^3^ cell/μL)	150-450	192	184
PCV (%)	35-47	40.6	38.5
Random blood glucose (mg/dL)	<125	132	94
Ureum (mg/dL)	10-59	45.4	64
Creatinine (mg/dL)	0.7-1.4	1.36	1.37
Sodium (mmol/L)	135-155	136	136.5
Potassium (mmol/L)	3.6-5.5	4.2	5.0
Chloride (mmol/L)	98-107	-	99.9
Troponin I (ng/L)	0-0.3	40,000	50,000

Urgent coronary angiography and primary percutaneous coronary intervention (PCI) were subsequently performed. Because of significant bradycardia related to CAVB, a PACE 101 H temporary transvenous pacemaker (Osypka Medical, Berlin, Germany) was inserted and set at a rate of 70 beats per minute, which effectively stabilized the ventricular rate prior to revascularization (Figure 2). Chest radiography confirmed appropriate lead placement without other acute abnormalities (Figure 3). Emergency coronary angiography revealed normal left main, left circumflex, and right coronary arteries. The culprit lesion was identified as a critical 95% stenosis at the ostial left anterior descending (LAD) artery with the presence of a thrombus (Figure 4). The lesion was pre-dilated using a 2.0 x 15 mm balloon. Subsequently, a 3.0 x 36 mm GuReater CoCr Sirolimus-eluting Coronary Stent System (Lepu Medical Technology (Beijing) Co., Ltd., Beijing, China) was successfully deployed from the ostial to mid-LAD artery at 15 atm. Post-PCI angiography confirmed excellent stent expansion with the restoration of TIMI grade 3 flow and no evidence of dissection.

**Figure 2:**
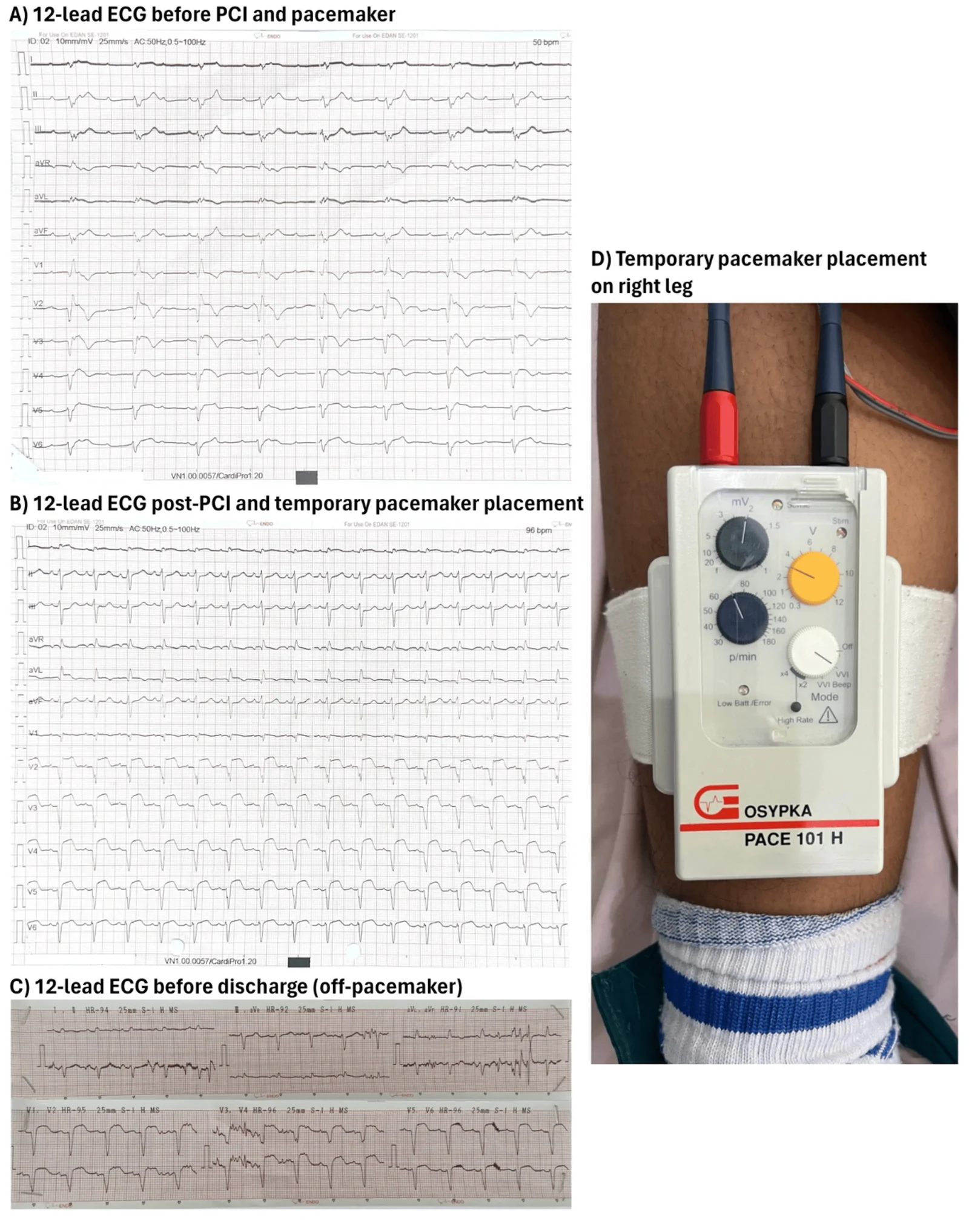
Serial electrocardiograms before and after primary percutaneous coronary intervention and temporary transvenous pacing.

**Figure 3:**
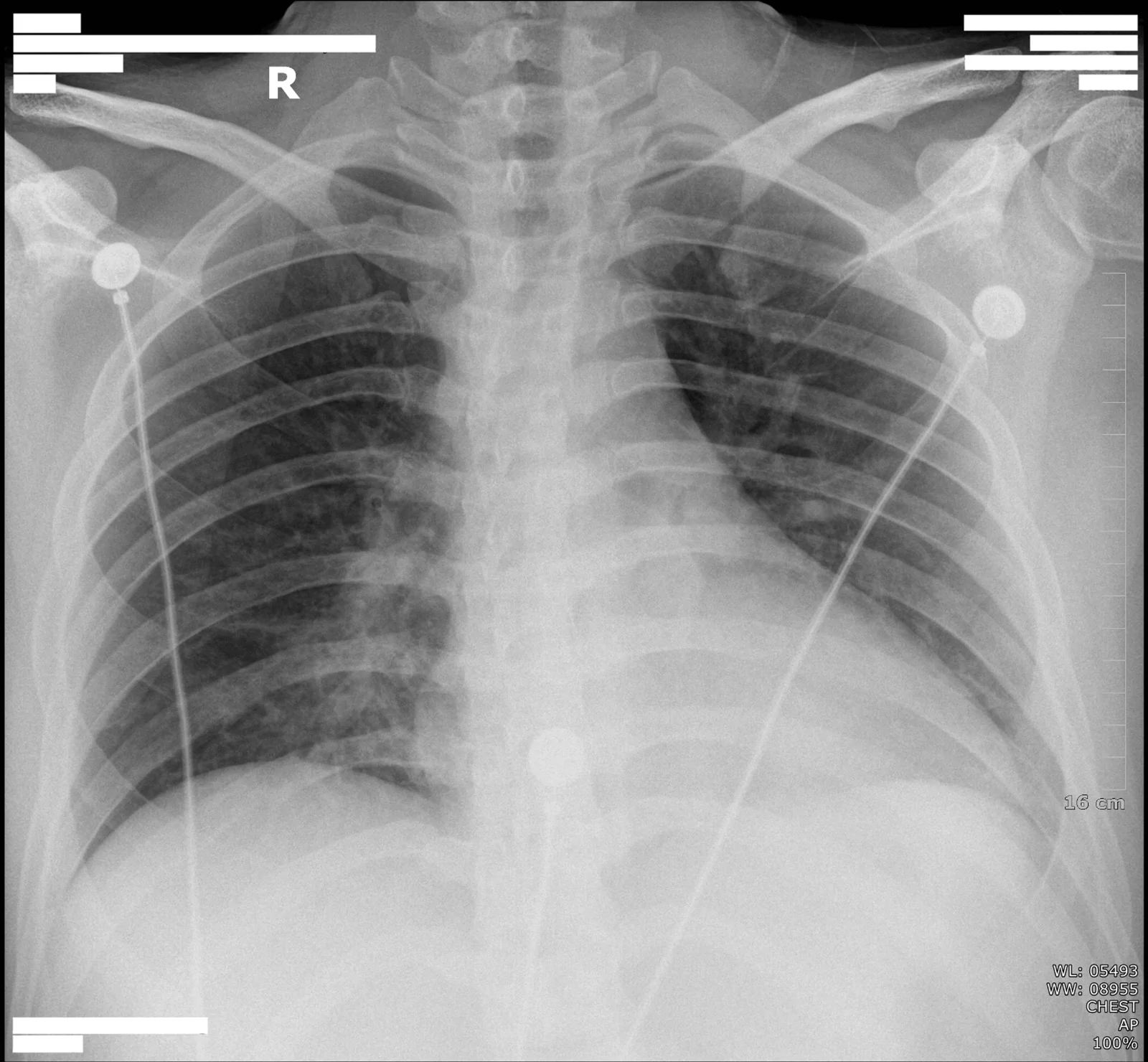
Chest X-ray following temporary pacemaker insertion showing appropriate lead positioning without acute abnormalities.

**Figure 4:**
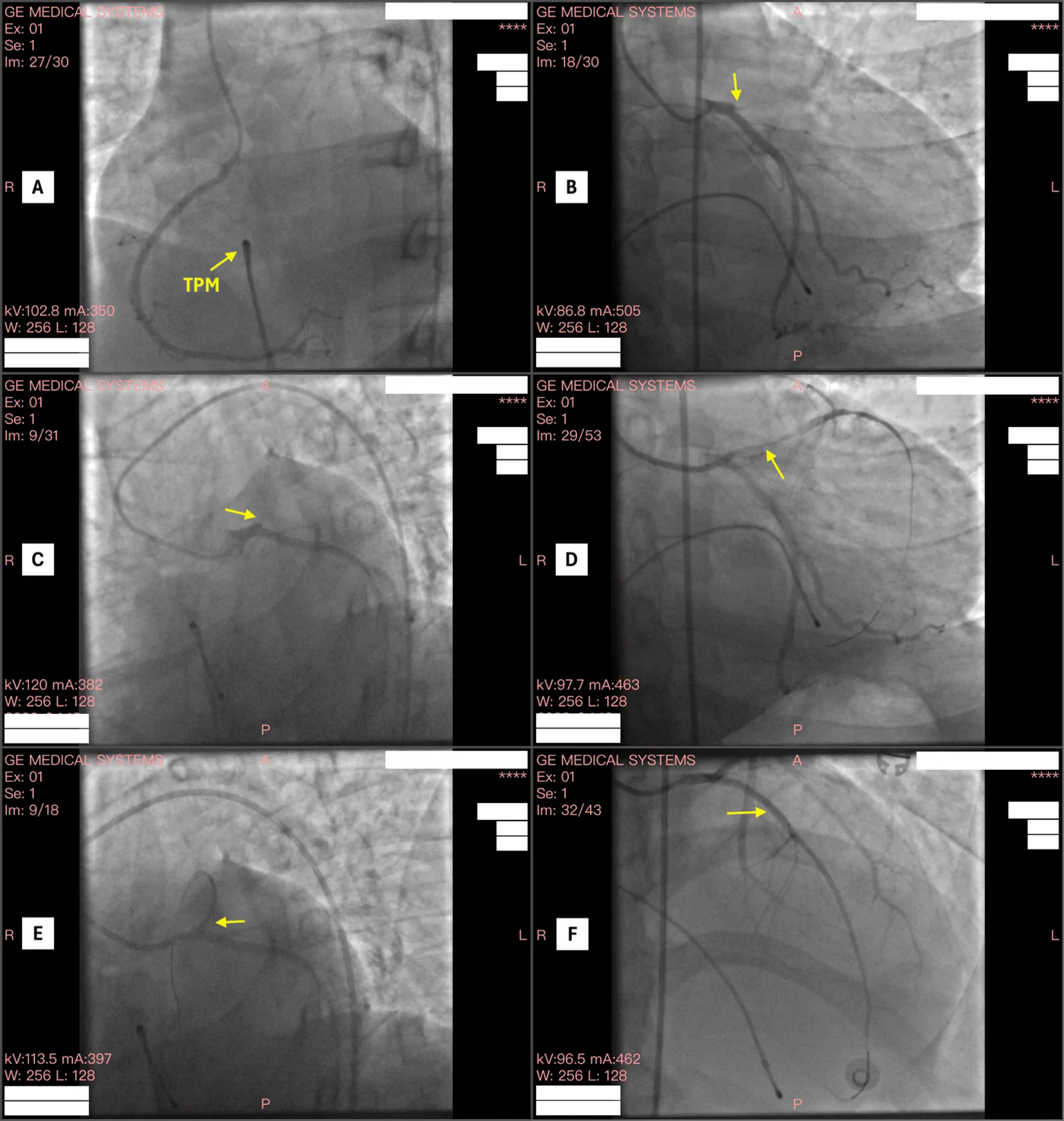
Serial coronary angiographic images during percutaneous coronary intervention (PCI) of the left anterior descending (LAD) artery. (A) Temporary pacemaker placement. (B–E) Sequential procedural angiographic images demonstrating intervention of the culprit LAD lesion with progressive restoration of the coronary flow. (F) Final angiogram showing improved LAD patency and distal coronary flow after PCI.

Following revascularisation, haemodynamic status improved rapidly, and atrioventricular conduction recovered, allowing removal of the temporary pacemaker without permanent pacing. The patient was discharged on the fourth hospital day and received counselling regarding cardiovascular risk-factor modification, particularly smoking cessation.

## Discussion

Acute myocardial infarction in young adults is relatively uncommon and may be overlooked because the presenting symptoms are not always typical [6-8]. In this case, the patient initially complained of epigastric pain, a recognised but often underappreciated presentation of anterior myocardial infarction [7]. As a result, the symptoms were initially considered gastrointestinal in origin, delaying ECG evaluation and definitive management [8]. The patient’s very young age likely also contributed to the delayed diagnosis, since acute coronary syndrome is often considered less likely in individuals in their early twenties without known cardiovascular disease [7,9].

Although myocardial infarction at this age is rare, young adults still account for approximately 2-10% of acute coronary syndrome cases, with a predominance in males [7,9]. In younger patients, modifiable risk factors, especially cigarette smoking, appear to play a more important role than diffuse multivessel atherosclerotic disease [6,7]. In our patient, smoking history, overweight status [10], and a family history of coronary artery disease [11] may have contributed to the event.

This case also suggests that acute coronary occlusion in very young individuals may occur even without widespread coronary disease. Cigarette smoking has been associated with endothelial dysfunction, platelet activation, and a prothrombotic state, all of which may promote acute thrombosis [7]. This mechanism may explain the sudden total occlusion of the LAD artery seen in our patient. Further investigations such as thrombophilia and autoimmune screenings or advanced vascular imaging were not performed; therefore, additional contributing factors cannot be completely excluded.

Delayed recognition in this patient was followed by progression to cardiogenic shock and CAVB. In anterior myocardial infarction involving proximal LAD occlusion, extensive septal ischemia may affect the conduction system and result in advanced atrioventricular block as well as haemodynamic instability. The accompanying RBBB further supported septal involvement. The rapid resolution of CAVB following primary PCI suggests that the infranodal conduction block in this patient was driven by transient severe ischemia rather than irreversible anatomical necrosis. Although permanent pacing is often required for persistent advanced block in anterior STEMI, our patient’s rapid conduction recovery allowed us to avoid permanent pacemaker implantation [12-14].

This report has several limitations. Previous studies reported a median hospital stay of approximately five days in comparable cases [13-15], whereas our patient was discharged on the fourth day after stable clinical improvement. Due to the hyperacute presentation with profound haemodynamic instability and Killip class IV cardiogenic shock, immediate life-saving primary PCI was prioritized, precluding a baseline transthoracic echocardiogram. Furthermore, a post-revascularisation echocardiogram and comprehensive lipid profile evaluations were not performed because of strict insurance limitations and the patient's early discharge following rapid clinical stabilization. The patient also declined further outpatient investigations.

In summary, this case demonstrates that severe myocardial infarction may still occur in very young individuals despite limited conventional cardiovascular risk factors. Early ECG evaluation and prompt reperfusion were associated with rapid recovery of haemodynamic status and atrioventricular conduction, eliminating the need for permanent pacemaker implantation in this patient.

## Conclusions

Myocardial infarction in very young adults may be difficult to recognise because the presenting symptoms are not always typical. Therefore, an atypical presentation in a young patient should not preclude early electrocardiographic assessment. In this patient, the initial complaint of epigastric pain was followed by rapid progression to cardiogenic shock and CAVB. Despite the severity of presentation, prompt coronary revascularisation was followed by the recovery of atrioventricular conduction. However, the avoidance of permanent pacemaker implantation was specific to this patient's rapid recovery following reperfusion, and decisions regarding permanent pacing in similar cases must remain individualized based on the clinical course.
